# Biclustering of transcriptome sequencing data reveals human tissue-specific circular RNAs

**DOI:** 10.1186/s12864-017-4335-9

**Published:** 2018-01-19

**Authors:** Yu-Chen Liu, Yu-Jung Chiu, Jian-Rong Li, Chuan-Hu Sun, Chun-Chi Liu, Hsien-Da Huang

**Affiliations:** 1 Institute of Bioinformatics and Systems Biology, National Chiao Tung University, Hsinchu, 300 Taiwan; 2 Institute of Genomics and Bioinformatics, National Chung Hsing University, Taichung, Taiwan; 3 Department of Biological Science and Technology, National Chiao Tung University, Hsinchu, 300 Taiwan; 4 Center for Bioinformatics Research, National Chiao Tung University, Hsinchu, 300 Taiwan; 5 Department of Biomedical Science and Environmental Biology, Kaohsiung Medical University, Kaohsiung, Taiwan; 6 Department of Biological Science and Technology, Institute of Bioinformatics, National Chiao Tung University, Hsinchu, 300 Taiwan, Republic of China

**Keywords:** Tissue specificity, circRNA, Biclustering

## Abstract

**Background:**

Emerging evidence has been experimentally confirmed the tissue-specific expression of circRNAs (circRNAs). Global identification of human tissue-specific circRNAs is crucial for the functionality study, which facilitates the discovery of circRNAs for potential diagnostic biomarkers.

**Results:**

In this study, circRNA back-splicing junctions were identified from 465 publicly available transcriptome sequencing samples. The number of reads aligned to these identified junctions was normalized with the read length and sequence depth for each sample. We generated 66 models representing enriched circRNAs among human tissue transcriptome through biclustering algorithm. The result provides thousands of newly identified human tissue-specific circRNAs.

**Conclusions:**

This result suggests that expression of circRNAs is not prompted by random splicing error but serving molecular functional roles. We also identified circRNAs enriched within circulating system, which, along with identified tissue-specific circRNAs, can serve as potential diagnostic biomarkers.

**Electronic supplementary material:**

The online version of this article (doi: 10.1186/s12864-017-4335-9) contains supplementary material, which is available to authorized users.

## Background

Circular RNAs (circRNAs) are a type of long non-coding RNAs, whose 3′ and 5′ ends joined into a single strand circular form. Although the existence of human circRNAs has been discovered and proven with electron microscopy for more than 30 years [1], it was only until 2012 with the advance of high throughput sequencing technology the ubiquitous expression of circRNA in mammals was found and proven [2]. Emerging evidence indicates the tissue-specific circRNAs play crucial roles in post-transcriptional level. Several cases of human circRNAs were found to serve as natural microRNA sponges [3, 4]. Biogenesis of circRNAs was found competing with the mRNAs of the host gene. In recent years, cell-free circRNAs were found in salvia and blood plasma [5, 6]. CircRNAs can potentially serve as diagnostic biomarkers for the undercover correlation to the pathogenesis of diseases and human physiological functions, as well as the stable circular forms. Global identification of human tissue-specific circRNAs is crucial for the study of circRNAs functionality.

The junctions between the 3′ and 5′ ends of the circRNAs have been referred as back-splicing junctions. The existence of circRNA within transcriptome sequencing data can be detected through identification of reads spanning these junctions. In previous studies [4], threshold applied to identify certain junctions as circRNA was that at least two unique reads spanning a head-tail junction. To discover human tissue-specific circRNAs, we collected 465 human transcriptome sequencing runs and applied the established pipeline. Expression level of circRNAs was estimated using the normalized counts of reads spanning the back-splicing junctions [7]. Biclustering [8] was conducted to detect circRNA expression patterns across different types of human tissues. From the result 66 bicluster models, we found a huge portion of circRNAs express only in the specific tissue type. This result suggests that expression of circRNAs is not prompted by random splicing error but serving molecular functional roles. We also identified circRNAs enriched within circulating system, which, along with identified tissue-specific circRNAs, can serve as potential diagnostic biomarkers.

## Results

**Fig. 1:**
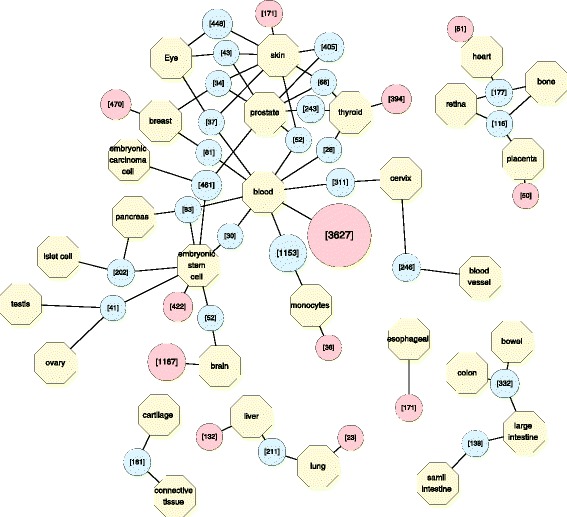
Network summary of the tissue-specific circRNAs. The circular nodes in this graph represents amount of grouped back-splicing junctions. Red nodes represent tissue-specific circRNAs, while blue nodes represent circRNAs enriched in multiple tissue types

A total 148,095 unique back-splicing junctions were identified from the selected transcriptome sequencing runs. Each of the junction site satisfy the threshold defined in the find_circ scripts [4, 9], as provided in the (Additional file 1). At least two reads were found spanning the identified site. More than 30000 junctions were found to have standard deviation of SRPBM among the 465 runs larger than 10. Through examining the alignment result with normalized value, we managed to find the tissue enriched circRNAs which would have been neglected. Back-splicing junctions with only one spliced read can be found in many datasets with high SRPBM values. The biclustering algorithm clustered 16,317 unique junctions into 66 coherent expression profile models (Additional file 2). The result of the biclustering reflects that the expression profiles of circRNAs are under tissue-specific regulation. A network view of the identified tissue-specific circRNAs is illustrated in Fig. 1.

### CircRNAs can be identified using poly A enriched RNA-Seq data

It had been assumed that since the exon originated circRNAs does not go through polyadenylation process after transcribed and spliced, they cannot be identified in the ploy-A enriched RNA-Seq data. However in several recent studies [2, 10, 11] circRNAs were identified in poly-A enriched RNA-Seq data sets, this could be due to the fact that some circular isoforms of the gene are adenine-rich. In this study, we discovered 24,589 unique back-splicing junctions from the 376 selected poly-A enriched RNA-Seq runs. One of the pivotal circRNA cdr1as [3, 4], which was proven to be nature miRNA miR-7 sponge, was found in 107 of our selected runs. Among these runs 71 are poly-A enriched.

### Novel back-spicing junctions

Compared with human circRNAs reported in 22 recent studies [4, 6, 7, 12–29], we found 5680 identified circRNAs back-splicing junctions has been reported in other studies. The remaining 92,015 unique back-splicing junctions are considered as novel circRNA candidates. Isoform annotation and the expression profiling can be found in the data base CircNet [30].

### Tissue-specific circRNAs

As illustrated in Fig. 1, the biclustering result provides thousands of tissue-specific expressed circRNAs. The nodes containing lower than 10 circRNAs, or connects to more than 3 types of tissues were hidden in the graph. The network demonstrates that circRNA co-expression profile following specific patterns similar to human genes [31]. Some groups of circRNAs express in multiple types of tissues with close correlated function. For example, the 332 circRNAs grouped with bowel, colon and large intestine might have potential physiological roles in the digest system, while the 243 circRNAs enriched in prostate and thyroid might correlate with male reproducing or development. The large amount of circRNA enriched in blood or blood cell samples suggests the ubiquity of circulating circRNAs, which makes circRNAs ideal diagnostic biomarkers. The tissue-specific circRNAs is available in (Additional file 3).

### Brain-specific circRNA host genes are enriched with synaptic GO terms

Based on the result of the gene-set enrichment analysis, we found that host genes of the brain-specific circRNAs are specially enriched with synapse-associated GO terms [32], as listed in Table 1. This result is consistent with the recent report regarding synaptic genes hosting circRNAs [33]. Through this study we hypothesize that these brain-specific circRNAs participate in the neuron development and synaptic functions. The back-splicing junction sites were enriched into these 9 gene groups, which can be found in (Additional file 4).

**Table 1:** Summary of the putative biomarkers

GO term	Genes	*P* value
GO:0043005|neuron projection	41	1.95E-24
GO:0045202|synapse	34	3.67E-17
GO:0030182|neuron differentiation	36	5.53E-16
GO:0042995|cell projection	44	1.73E-15
GO:0044430|cytoskeletal part	48	2.92E-13
GO:0030424|axon	20	3.54E-12
GO:0031175|neuron projection development	24	7.24E-12
GO:0048666|neuron development	27	1.06E-11
GO:0015630|microtubule cytoskeleton	34	1.09E-11

The GO term enrichment of back spliced junction sites clustered into brain is summarized in this table

### Potential diagnostic biomarkers revealed from the results of the biclustering

Besides the tissue specificity of human circRNAs, putative diagnostic biomarkers can be discovered from the bicluster models. We found 607 back-splicing junctions and cancer skin/prostate samples were clustered into the same bicluster (SD10_10.txt from Additional file 2). Samples with close conditions originated from the same tissue types were biclustered with back-spicing junctions, suggesting that circRNAs originated from these back-splicing junctions express specifically to the disease condition as well as tissue type. With sufficient experiment verification as well as population studies, these circRNAs can serve as potential diagnostic biomarkers. These back-splicing junction sites and the related conditions are summarized in Table 2. A full list of these biomarkers is available in (Additional file 5).

**Table 2:** Summary of the putative biomarkers

# junctions	Tissue	Condition	Model
607	Skin	Cancer	SD10_10.txt
457	Breast	Cancer	SD10_33.txt
589	Blood	Cancer	SD10_29.txt
625	Blood	LVAD placement	SD10_31.txt
476	Cervix	Cancer	SD10_8.txt
577	Brain	Normal	SD10_34.txt

The number of back-splicing junctions clustered into tissue and condition models is summarized in this table

## Discussion

In this study, we identified the potential tissue specific circRNA through conducting biclustering on expression profiles of circRNA across multiple human tissue samples. Despite the promising results, several limitations are worth-mentioning.

First of all, RNA-Seq data set collected in this study are retroactive sourced. Potential Batch effect was inevitable. Expression profiles within poly-A enriched samples were also biased. On the other hand, the expression profile was based on the normalized count of back spliced junction site spanning reads. Without accurate annotation of the full-length sequence of circRNA, this kind of measurement can be limited. Finally, the gene set enrichment conducted in this study was based on the genes locus that intersect with back spliced junction sites. This analysis was based on the assumption that functions of circRNAs correlate with the functions of back spliced junction sites overlapped genes. Weather tissue specific genes correlate with the biogenesis of tissue specific circRNA Is also an ongoing research subject. Further analysis of the issue specific circRNA locus correlation with known tissue specific genes should be conducted in the near future.

## Conclusions

From the result 66 bicluster models, we found a huge portion of circRNAs express only in the specific tissue type. This result suggests that expression of circRNAs is not prompted by random splicing error but serving molecular functional roles. We also identified circRNAs enriched within circulating system, which, along with identified tissue-specific circRNAs, can serve as potential diagnostic biomarkers after sufficient experiment verification as well as population studies.

## Methods

**Fig. 2:**
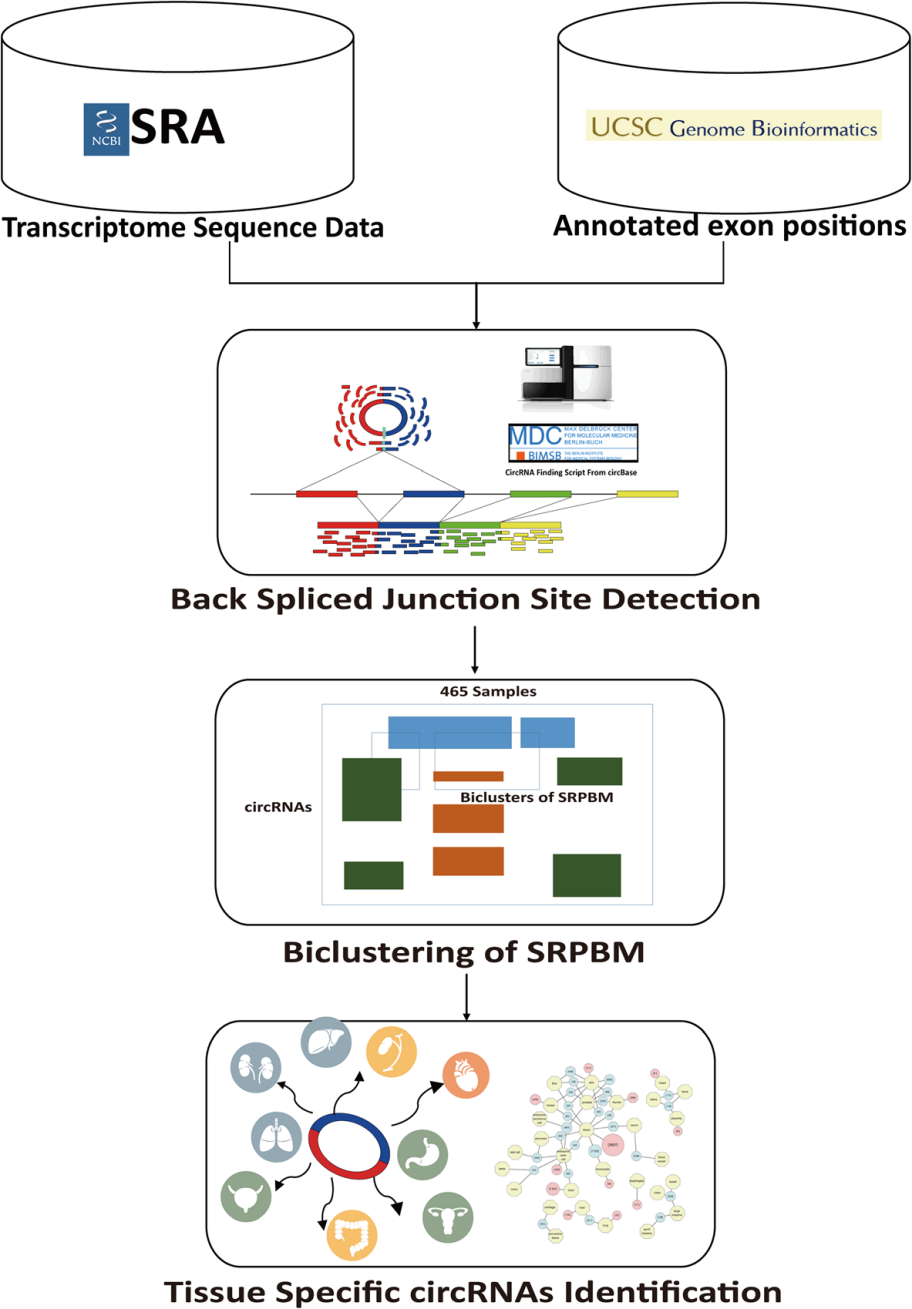
Summary of the data analysis process. The data analysis process conducted in this study is summarized in this flowchart

As illustrated in Fig. 2, 465 RNA-Seq runs were collected from a wide range of independent experiments across 26 human tissues and 104 disease conditions from the NCBI Sequence Read Archive [34], in which 89 runs are non-polyA enriched RNA-Seq data. Selection of the RNA-Seq runs was made on purpose of covering as many different conditions as possible. Quality control of the sequence reads was conducted through the NGS QC toolkit [35] with default setting. The algorithm referred as find_circ [4, 9] was applied to detect back-splicing junctions. To normalize the amount of the normalized sequence reads spanning the junctions, a concept of spliced reads per billion mapping (SRPBM) was applied [7]. Amount of reads mapped onto hg19 human genome was acquired through the tool STAR [36]. The equation applied to calculate SRPBM is:

1 $$ SRPBM=\frac{Read s count\times {10}^9}{Read lenth\times Mapped reads} $$

The junction sites with standard deviation of SRPBM among the 465 runs larger than 10 were further selected for biclustering analysis. For searching the coherent expression profiles. The R package ‘isa2’ [37] was used for Iterative Signature Algorithm analysis. Coherent expression pro-files of selected 30000 junctions among the 465 runs was acquired from the bicluster models generated from Iterative Signature Algorithm [38]. A network analysis was conducted on the grouped junctions, and the network was illustrated through Cytoscape [39]. Gene sets enrichment of the circRNA host genes was conducted through DAVID [40]. Back spliced junction sites clustered into models containing only one types of tissue were considered as tissue-specific circRNAs originated.

## Additional files

Additional file 1: One hundred forty-eight thousand ninety-five unique back-splicing junctions were identified from the selected transcriptome sequencing runs. Each of the junction site satisfy the threshold defined in the find_circ scripts. (XLSX 10182 kb)

Additional file 2: The biclustering algorithm clustered 16,317 unique junctions into 66 coherent expression profile models. (RAR 1442 kb)

Additional file 3: The tissue-specific circRNAs. (XLSX 426 kb)

Additional file 4: The back-splicing junction sites were enriched into these 9 gene groups. (TXT 133 kb)

Additional file 5: Collection of circRNAs can serve as potential diagnostic biomarkers. (XLSX 110 kb)
